# Editorial: Pathways for Rapid Visual Processing: Subcortical Contributions to Emotion, Threat, Biological Relevance, and Motivated Behavior

**DOI:** 10.3389/fnbeh.2022.960448

**Published:** 2022-06-27

**Authors:** Robin Laycock, John F. Stein, Sheila G. Crewther

**Affiliations:** ^1^School of Health and Biomedical Sciences, RMIT University, Melbourne, VIC, Australia; ^2^School of Psychology and Public Health, La Trobe University, Melbourne, VIC, Australia; ^3^Department of Physiology, Anatomy and Genetics, University of Oxford, Oxford, United Kingdom; ^4^Centre for Human Psychopharmacology, Swinburne University of Technology, Melbourne, VIC, Australia

**Keywords:** visual, subcortical, emotion, fear, autism, schizophrenia

From birth, the visual system is the predominant sensory system that provides information for primate and human understanding, visuomotor actions, emotion, cognition and interaction with the outside world. Whether it be reading a book or understanding the social cues of a conversation partner, these skills are first of all visual. The last 40 years of vision science have provided incredibly detailed knowledge, mainly focussing on the primary more conscious geniculo-striate pathway. However, the eye and the retina also project to the brain stem superior colliculus, and many other thalamo-cortical regions apart from the primary visual cortex. These subcortical shortcuts are critical for rapid alerting of attention, eye-movements, and non-conscious (or pre-conscious) emotional responses, and form an important focus of this Research Topic.

Animal studies have provided seminal evidence of the anatomical and physiological properties of these visual networks (e.g., Benevento and Standage, 1983; Maunsell and Gibson, 1992). In this special issue Chen et al. used retrograde and anterograde tracing methods in rat to reveal the multimodal inputs to area prostriata, a previously under-explored region. Inputs were received from sensory cortical and also subcortical regions. Interestingly a short-cut pathway from the dorsal LGN was demonstrated by Chen et al., likely subserving fast motion perception in peripheral vision, and as the authors suggest, may be important for explaining unconscious perception in blindsight. Outputs of area prostriata include a range of subcortical regions that also highlight how rapid visuomotor behaviors are driven, potentially having implications for rapid and potentially adaptive responses to unexpected looming threats in the environment.

Although brain structures associated with the “social brain” such as the amygdala have traditionally been conceptualized as being activated *via* the slower cortical visual pathways from V1 much of the more recent marmoset work (Linke et al., 1999; Day-Brown et al., 2010) has demonstrated that the amygdala is activated by a more direct route through superior colliculus and pulvinar. Such a system, which has also been demonstrated in human (Méndez-Bértolo et al., 2016; McFadyen et al., 2019) would be expected to be faster and foster concurrent benefits in being able to rapidly activate autonomic and emotional processes potentially prior to conscious deliberation. This pathway is also strongly implicated in fast, automatic, and even non-conscious emotional processing of faces and potential threats (Tamietto and de Gelder, 2010).

In this issue, Dinh et al. contribute to this literature using single-unit recording from amygdala in Japanese Macaques. They examined rate of neuronal responses to images of natural primate predators (snakes, raptors and carnivores), non-predator animals, human and monkey faces and found shorter latency (<100 ms) and larger amplitude to snakes than other primate predators such as raptors and carnivores, while emotional faces also produced faster and stronger responses than neutral faces. Responses to emotional faces were also stronger for low-pass than high-pass filtered images. Overall, the results suggest that the amygdala facilitates rapid processing of threatening and novel stimuli. Dinh et al. also found response magnitude of amygdala neurons correlated with pulvinar responses collected in earlier work with the same stimuli (Le et al., 2013), further highlighting the functional role of a subcortical pathway through superior colliculus, pulvinar and the amygdala.

This Research Topic also sought to further understanding of the contribution of subcortical and thalamo-cortical visual pathways in driving fast processing–in infants and in adults–with a particular focus on emotional processing in healthy populations as well as in neurodevelopmental and psychiatric disorders.

Neurodevelopmental or psychiatric disorders are of particular interest, with difficulties in social cognition often linked to amygdala dysfunction (e.g., Peng et al., 2020). In this issue Martinez et al. have shown how face emotion recognition deficits in ASD and schizophrenia could be explained by different pathophysiology. Adults with schizophrenia or ASD both underwent fMRI and demonstrated deficits in STS activation though impaired activation in V1 and motion sensitive regions was only observed in schizophrenia, with STS activation intercorrelated with early visual cortex and pulvinar activation. Indeed, a mediation analysis revealed a pathway from V1 to STS, *via* the medial subdivision of the pulvinar. On the other hand, ASD was associated with increased V2 and prefrontal responses. While overall pulvinar activation was lower for schizophrenia compared to both ASD and control groups for emotional faces, further analysis confirmed this effect for lateral pulvinar (that receives large input from V1 (Purushothaman et al., 2012) and plays a large role in consciousness) and medial subdivisions, while the ASD group only showed higher activation in the inferior pulvinar compared to schizophrenia and control groups. Thus, Martinez et al. argue that integration between subcortical and cortical visual pathways are important for social cognition, and abnormalities in these circuits are important for complex neurocognitive disorders.

How anxiety influences activation and reliance on these phylogenetically older pathways is also of relevance. McFadyen et al. examined how prediction errors for neutral and fearful faces influence perceptual decision making, when employing a paradigm that enables previously unconscious stimuli to emerge into conscious awareness when continuous flash suppression (CFS) is broken. As expected, perceptual discrimination was faster for fear than neutral faces, and slower for unexpected compared with expected neutral faces. Interestingly, it was also shown that responses were faster for unexpected compared with expected fearful faces, though this effect was driven by high anxiety participants. These opposing effects of expectancy on emotion expression was also reflected in initial sensory encoding in the EEG. McFadyen et al. argue for a “survival hypothesis” in which biologically relevant stimuli (i.e., fearful faces in this case) are given priority access to conscious awareness, particularly when unexpected, and more-so for those with higher anxiety. Thus, neural mechanisms appear capable of differentially prioritizing expected and unexpected stimuli depending on the biological relevance.

The clinical relevance of understanding subconscious processing was further examined by Frumento et al. in the context of specific phobia. The authors provide a systematic review concluding that subconscious presentation of phobic stimuli can result in extinction of the phobic response, without the typical subjective distress associated with standard exposure therapies involving the conscious experience of phobic stimuli. The authors go on to propose a new therapeutic model for treatment of phobias, integrating both supra and subliminal exposure, given these appear to involve separate systems.

While the anatomy and physiology of the primate visual system is well established through a number of cat and monkey studies in the 1980's and 90's (for reviews, see Laycock et al., 2007; Nassi and Callaway, 2009)], Spiteri and Crewther in this issue provide a useful update based on more recent monkey research over the last 20 years. This update culminates in an intriguing new hypothesis to explain visual anomalies in the autism spectrum. The focus is on the developmental course of the dorsal cortical stream, and how an inferior pulvinar to MT pathway is developmentally superseded by a geniculate-striate pathway to MT. Spiteri and Crewther propose that a developmental slowing of the withdrawal of the pulvinar-MT connection in autism, driven by abnormal amygdala direct connections to the thalamic reticular nucleus than surrounds the thalamus, enhances visual responsiveness through the pulvinar. The proposal already has some evidence, as outlined in the review, while some important questions for further research are suggested.

This special issue has explored only some of the adaptive and clinical implications of these visual circuits outside the main geniculo-striate pathways. It is hoped that these articles help to highlight the continuing research on the visual system's role in facilitating emotional and otherwise salient information processing.

## Author Contributions

RL wrote the initial manuscript. All authors contributed to the article and approved the submitted version.

## Conflict of Interest

The authors declare that the research was conducted in the absence of any commercial or financial relationships that could be construed as a potential conflict of interest.

## Publisher's Note

All claims expressed in this article are solely those of the authors and do not necessarily represent those of their affiliated organizations, or those of the publisher, the editors and the reviewers. Any product that may be evaluated in this article, or claim that may be made by its manufacturer, is not guaranteed or endorsed by the publisher.
